# Implicit Grasp Force Representation in Human Motor Cortical Recordings

**DOI:** 10.3389/fnins.2018.00801

**Published:** 2018-10-31

**Authors:** John E. Downey, Jeffrey M. Weiss, Sharlene N. Flesher, Zachary C. Thumser, Paul D. Marasco, Michael L. Boninger, Robert A. Gaunt, Jennifer L. Collinger

**Affiliations:** ^1^Department of Bioengineering, University of Pittsburgh, Pittsburgh, PA, United States; ^2^Center for the Neural Basis of Cognition, Pittsburgh, PA, United States; ^3^Department of Organismal Biology and Anatomy, University of Chicago, Chicago, IL, United States; ^4^Department of Physical Medicine and Rehabilitation, University of Pittsburgh, Pittsburgh, PA, United States; ^5^Department of Neurosurgery, Stanford University, Stanford, CA, United States; ^6^Laboratory for Bionic Integration, Department of Biomedical Engineering, Lerner Research Institute, Cleveland Clinic, Cleveland, OH, United States; ^7^Research Service, Louis Stokes Cleveland VA Medical Center, Cleveland, OH, United States; ^8^Advanced Platform Technology Center of Excellence, Louis Stokes Cleveland VA Medical Center, Cleveland, OH, United States; ^9^VA Pittsburgh Healthcare System, Pittsburgh, PA, United States

**Keywords:** brain-computer interface, neuroprosthetics, motor cortex, intracortical, grasp force

## Abstract

In order for brain-computer interface (BCI) systems to maximize functionality, users will need to be able to accurately modulate grasp force to avoid dropping heavy objects while also being able to handle fragile items. We present a case-study consisting of two experiments designed to identify whether intracortical recordings from the motor cortex of a person with tetraplegia could predict intended grasp force. In the first task, we were able classify neural responses to attempted grasps of four objects, each of which required similar grasp kinematics but different implicit grasp force targets, with 69% accuracy. In the second task, the subject attempted to move a virtual robotic arm in space to grasp a simple virtual object. For each trial, the subject was asked to grasp the virtual object with the force appropriate for one of the four objects from the first experiment, with the goal of measuring an implicit representation of grasp force. While the subject knew the grasp force during all phases of the trial, accurate classification was only achieved during active grasping, not while the hand moved to, transported, or released the object. In both tasks, misclassifications were most often to the object with an adjacent force requirement. In addition to the implications for understanding the representation of grasp force in motor cortex, these results are a first step toward creating intelligent algorithms to help BCI users grasp and manipulate a variety of objects that will be encountered in daily life.

**Clinical Trial Identifier:** NCT01894802 https://clinicaltrials.gov/ct2/show/NCT01894802.

## Introduction

Brain-computer interfaces (BCI) have shown promise as assistive devices to restore a level of independence to people with tetraplegia (Collinger et al., 2012, 2013; Hochberg et al., 2012; Wodlinger et al., 2014; Blabe et al., 2015; Ajiboye et al., 2017). However, one significant limitation of the work to date is that control is limited to the kinematic domain where only the position or speed of the arm and hand are controlled (Collinger et al., 2012; Hochberg et al., 2012; Wodlinger et al., 2014). To achieve independence, users will need to modulate grasp force to maintain stable grasp postures and to handle objects that vary in weight, compliance, or fragility. Additionally, with the recent demonstration that intracortical microstimulation in somatosensory cortex can convey graded tactile percepts from many different locations on the hand (Flesher et al., 2016), it is now possible to provide feedback about grasp force through the BCI.

There is evidence from rats (Khorasani et al., 2016), non-human primates (Maier et al., 1993; Hepp-Reymond et al., 1999; Hendrix et al., 2009), and human subjects (Flint et al., 2014; Murphy et al., 2016) that grasp force-related information can be recorded from primary motor cortex (M1). Here we explore the representation of implicit grasp force from extracellular recordings in M1 of a single BCI user with tetraplegia, who is physically unable to generate overt grasping movements. We evaluate whether the visual presentation of objects of varying compliance and weight can elicit discriminable patterns of activity. We also investigate whether grasp force-related information is present during a multi-phase object transport task where no visual feedback about object identity is provided. During attempted grasping movements, M1 recordings clearly discriminated between objects with different force requirements, though this information was not present during reaching and object transport.

## Materials and Methods

This study was conducted under an Investigational Device Exemption from the U.S. Food and Drug Administration for this clinical trial (NCT01894802). The study was approved by the Institutional Review Boards at the University of Pittsburgh and the Space and Naval Warfare System Center Pacific. All procedures were conducted in accordance with the policies and guidelines associated with these approvals.

### Subject

A 28 year-old male with C5 motor/C6 sensory ASIA B spinal cord injury provided written informed consent prior to participation. He had two 4 × 4 mm, 88-channel microelectrode arrays (Blackrock Microsystems, Salt Lake City, UT, United States) implanted in the hand and arm area of M1 in the left hemisphere (Locations shown in Figure 1C of Flesher et al., 2016). He also had two microelectrode arrays implanted into somatosensory cortex for stimulation that were not used in this experiment (Flesher et al., 2016).

### Neural Recording

Extracellular potentials were hardware filtered between 0.3–7500 Hz and sampled at 30,000 Hz, then digitally high-pass filtered at 750 Hz with a first order Butterworth filter. At the beginning of each test session the spike threshold was set to −4.5 times the root-mean-square voltage on each channel. The number of threshold crossings on each channel were binned in 20 ms increments.

### Attempted Grasp vs. Object Observation

**FIGURE 1:**
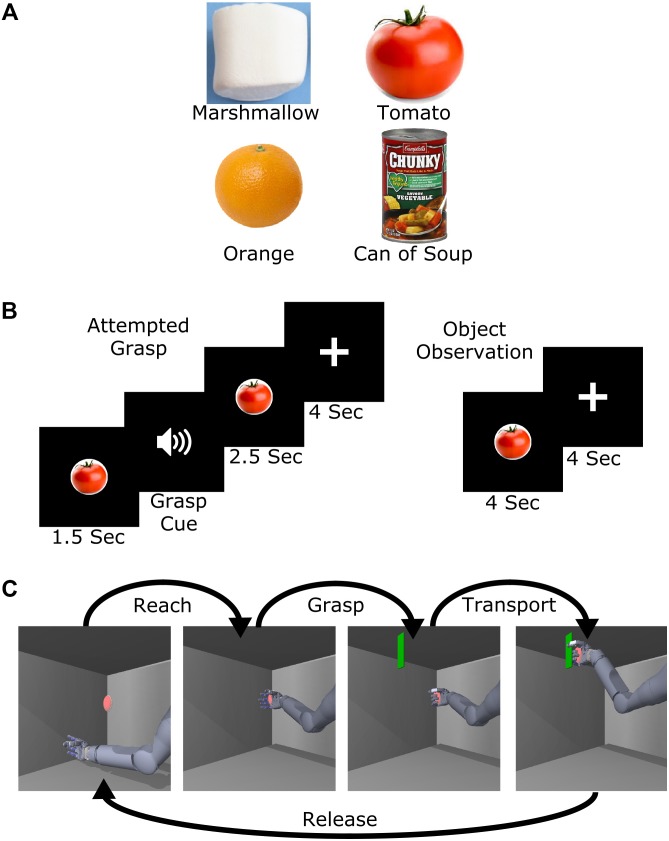
Experimental design. **(A)** Images of the four objects selected to represent different levels of required grasp force. **(B)** Task presentation and timing of the attempted grasp and object observation trials. **(C)** Object transport trial example. For this task, the name of one of the four objects was played at the start of the trial and the subject was then asked to attempt to use the virtual arm to grasp and transport the virtual object with the appropriate amount of force. Five position targets were used including a center target and four others approximately 20 cm above, below, left, and right of center.

The subject was asked to watch a television screen where images of four different objects were presented: a large marshmallow, a tomato, an orange, and a can of soup (Figure 1A). These objects were chosen in consultation with the subject to have varying levels of compliance and weight, which resulted in evenly spaced perceived levels of grasp force required to lift them. These objects were chosen, as opposed to explicitly named force levels, because previous work has shown that implicit force targets lead to more consistent grasp force exertion in able-bodied subjects (Thumser et al., 2018). The objects were approximately the same size, and the subject was instructed to attempt a 5-finger power grasp for all objects, even though his hand was paralyzed, in an attempt to keep grasp kinematics consistent. For all trials, the target object was shown for 4 s, followed by 4 s of rest during which a fixation cross was displayed before the next trial started (Figure 1B). During attempted grasp trials, an audio cue was played 1.5 s after the object appeared to instruct the subject to attempt to grasp the object with the amount of force necessary to lift the object without crushing it. This strategy of attempting a movement even when no overt movement is generated is commonly used for BCI calibration (Taylor et al., 2002; Velliste et al., 2008; Collinger et al., 2012). During object observation trials, the subject was instructed to simply observe the objects without attempting to grasp. Across three test sessions (332, 372, and 378 days post-implant), 132 trials (33 per object) of both attempted grasp and object observation conditions were collected.

### Object Transport Task

To study implicit grasp force representation in the context of whole arm movement, we adapted a virtual reality task (Wodlinger et al., 2014) where the arm moved in a two-dimensional (2D) vertical plane with a single grasp dimension (flexion or extension of all fingers). The task consisted of a sequence of reaching to, grasping, transporting, and releasing a virtual object. The arm was moved automatically while the subject attempted to make the movements, as occurs during BCI calibration (Collinger et al., 2012; Wodlinger et al., 2014). At the start of each trial, one of the four objects (Figure 1A) was spoken as a computer-generated audio cue. Next, a red ellipsoid appeared at a target position (Figure 1C). Once the hand reached the ellipsoid (reach phase), an audio cue prompted the subject to attempt to grasp the object (grasp phase). The instructions to the subject were to attempt a 5-finger power grasp of the virtual object with the amount of force appropriate to hold the spoken object without letting it slip, even though his hand was paralyzed. A green plane then appeared and the subject attempted to move the ellipsoid to that location (transport phase). Finally, an audio cue instructed the subject to release the ellipsoid (release phase). The subject completed two sessions (374 and 576 days post-implant) of 99 trials each for a total of 198 trials.

### Classification of Object-Related Grasp Force

To determine whether implicit grasp force was represented in M1 during attempted grasp and object observation, we performed naïve Bayes classification (Bishop et al., 2014; Perge et al., 2014) on 1 s of neural data starting 2 s after the start of the trial (Figure 1B). Thresholded channels with average firing rates higher than 2.5 Hz were used for classification; on average 125 channels were used for classification from each session. For attempted grasp and observation trials, the 1 s window started 2 s after the object was presented (0.5 s after the grasp cue in grasp trials). This time period was selected based on a preliminary analysis showing chance-level classification prior to attempted grasp, with classification accuracy increasing during the 500 ms after the grasp cue. For cross-validation, we left out one random trial from each class (i.e., object type) during classifier training and then tested the classifier on the left-out trials, repeating until all trials had been left out. This process was repeated five times for each session to get multiple combinations of left-out trials. We estimated the 95% confidence intervals of chance level classification by shuffling the object labels and repeating the classification process 500 times.

We performed the same classification on neural data from the grasp and transport phases of the object transport task. We used 157 channels for classification in session 1 and 131 channels in session 2. For the grasp phase, the last 1 s of data were used for classification. For the transport phase, we selected 1 s of active reaching, which ended 500 ms before the end of the phase.

We further examined how classification accuracy evolved over the duration of object transport trials using a sliding window of neural data. Since the reach and transport phases were different lengths across trials, data were aligned to the grasp and release cues. A 500 ms trailing window with 100 ms step sizes was used to classify grasp force level for 1.6 s before and 0.8 s after the grasp and release cues. Confidence intervals on chance level performance were estimated for each classification window using shuffled object labels as described above.

## Results

### Object Observation vs. Attempted Grasp

**FIGURE 2:**
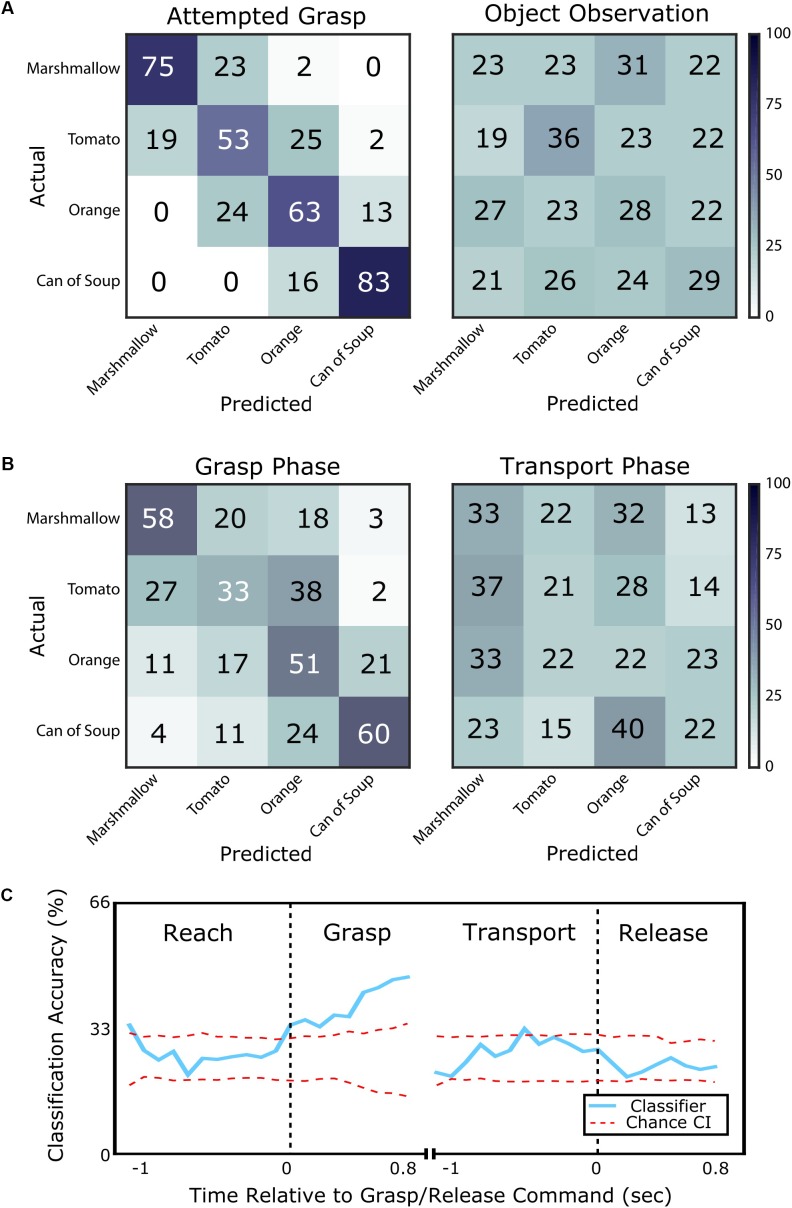
Classification of object identity for objects of various compliances and grasp force requirements. **(A)** Confusion matrices showing classification accuracy for each object from the attempted grasp vs. object observation task. Each row shows the percentage of times the corresponding object was predicted to be each of the four possible objects (numbers may not add to 100 due to rounding). On the left, classification of the presented object is accurate (69% success) when the subject is attempting to grasp it. Errors are typically to adjacent objects in terms of compliance and required grasp force. On the right, classification of the presented object when the subject simply observes the object is within chance and classification errors are randomly distributed. **(B)** Confusion matrices showing classification accuracy from the grasp and transport phases of the object transport task. On the left, classification is accurate (50% success) during the grasp phase with errors typically to an adjacent object in terms of required grasp force. On the right, classification during the transport phase is near chance and classification errors are randomly distributed. **(C)** Classification accuracy computed for the object transport task, shown as the blue line aligned to the cue to grasp (left) and release (right). The red dotted lines bound the 95% confidence interval on chance performance. Classification is above chance during the grasp phase, but is generally within chance level for the other phases of movement.

The subject, who was unable to move his own hand voluntarily, was asked to attempt to grasp visually presented objects (marshmallow, tomato, orange, or can of soup) with an appropriate amount of force to lift them. We could predict with 69% accuracy which object he was attempting to grasp using 1 s of neural data starting 500 ms after the grasp cue. This was significantly better than the chance rate of 25 ± 9% (95% confidence interval). Additionally, 97% of misclassified trials were predicted to be an object with an adjacent implicit grasp force level (95% confidence bounds on chance: 51 ± 10%, Figure 2A). When the subject simply observed the objects, but did not attempt to grasp, classification accuracy was 29%, which was no better than chance (95% confidence bounds on chance: 25 ± 9%).

### Object Transport Task

We also examined whether grasp force information could be classified in a more complex task where the subject attempted reaching to, grasping, and transporting an object with an appropriate amount of force. During the grasp phase, object type could be predicted with 51% accuracy and 73% of misclassifications fell within one force level of the target (Figure 2B). However, classification accuracy during the transport phase was only 25%, which was no better than chance. During transport, only 56% of misclassifications were within one force level of the target.

A sliding window classifier revealed the time course of classification accuracy. This analysis used 500 ms of neural data as compared to 1 s for the classification results presented above. During the reach phase, object identity could only be classified at chance level (26%). However, over the course of the grasp phase, classification accuracy improved from 34% at grasp phase onset to 47% 800 ms into the grasp phase (95% confidence bounds on chance: 25 ± 7%, Figure 2C). During the transport phase, classification accuracy returned to chance level (27%). The subject confirmed that he continued to attempt to grasp the object with the appropriate force during the transport phase. Finally, during the release phase, average classification accuracy remained at chance level (23%).

## Discussion

Here we show that implicit grasp force is represented in M1 during attempted grasp, but not during observation, of objects of varying compliance, weight and texture. Importantly, implicit force was not well-represented in M1 during periods where the force was maintained, such as transport, or when the object was released. While previous studies identified grasp force information in EEG signals (Rearick et al., 2001; Murguialday et al., 2007; Paek et al., 2015; Wang et al., 2017) and intracortical field potentials of motor-intact subjects (Flint et al., 2014; Murphy et al., 2016), here we show that this information is present in multi-unit recordings of a subject with tetraplegia. To identify information about implicit grasp force in a subject who could not generate grasp forces, we asked him to attempt to grasp objects that he was familiar with grasping before his injury. Our result that M1 contains implicit information about grasp force aligns well with data from able-bodied subjects showing that the use of everyday objects as implicit grasp force cues led to a more consistent separation of executed grasp forces during the initial dynamic phase of the grasp as compared to trying to match a visual force target (Thumser et al., 2018).

We observed that implicit grasp force was not well-represented in M1 during object transport, which is consistent with a prior report that grasp force encoding is context dependent (Hepp-Reymond et al., 1999). However, it is inconsistent with reports that more neurons were modulated during static grasp force exertion than during the initial application of grasp force (Mason et al., 2002). One possibly important difference between the Mason et al. (2002) study and ours is that their work did not include object transport while grasping, which could change the context of the coding in M1. Another difference is that Mason et al. (2002) used single electrode recordings in multiple sites of a large window spanning M1 and dorsal premotor cortex compared to our microelectrode array recordings from a smaller, fixed location. This difference could have resulted in Mason et al. (2002) recording from a more heterogenous population of neurons, including corticomotor neurons that make monosynaptic connections to the motoneurons innervating the muscles in the hand and forearm (Rathelot and Strick, 2009). Identifying the cause of this change in coding between movement phases will require additional experiments.

Our study design required the participant to attempt to grasp familiar objects in order to elicit an implicit representation of grasp force even though no overt movement was performed due to his paralysis. The subject was instructed to use consistent grasp kinematics to pick up the objects without deforming them, however, there was no way to measure his attempted kinematics because he could not move his own hand. Because the movements were performed covertly, there are limitations to the interpretation of the results. We were unable to measure the impact that differences in the visualized kinematics or object properties, such as texture or compliance, had on the neural activity. The objects were chosen to be equally spaced within the subject’s reported implicit force range. During attempted grasping of these objects, nearly all classification errors were to an object that was directly adjacent to the target object on the implicit force spectrum. This is despite the fact that the two cylindrical objects that were most similar in shape (large marshmallow and can of soup) were at opposite ends of the force spectrum. Previous work by Schaffelhofer et al. (2015) showed that cylindrical objects of differing sizes, like our marshmallow and can of soup, were more likely to be confused with each other by a classifier using recordings from M1 than with spheres, like our tomato and orange, of any size when primates planned and executed movements to a large variety of different sized and shaped objects. The systematic classification prediction error in our results suggests that implicit grasp force is represented in M1 in an intuitive way.

The familiar objects used in this study differ in terms of visual appearance and expected sensory consequences (e.g., texture and compliance). If the visual and sensory properties of the objects were the primary driver of neural activity, we would have expected misclassification errors to be more randomly distributed. The decision to use familiar objects was motivated by previous work in able-bodied subjects that found that grasp forces were applied more consistently when presented with familiar objects as compared to being instructed to hit explicit force targets on a single object (Thumser et al., 2018). It is also important to note that M1 activity was modulated during the grasp phase of the object transport task, even though the object being grasped remained visually the same for all trials (i.e., a red ellipsoid). This provides additional evidence that planning for slight differences in grasp kinematics or finger placement is not a primary driver of classification accuracy.

We leveraged the implicit neural representation of grasp force for familiar objects to identify grasp force information in M1. The classification accuracies reported here, while above chance, will need to improve to provide reliable BCI control and give the user continuous control over grasp force. Additionally, the BCI will need information about grasp kinematics or state (e.g., open/close). Further work is necessary to optimally extract this information and determine whether this representation of grasp force generalizes well to other objects. Based on this work, we believe that decoders will need to include intelligent algorithms that can recognize when grasp force information is available in M1 and then exert that force through the prosthesis at the appropriate times during object manipulation. Similarly, for BCIs that aim to restore native limb movement through muscle stimulation, one could modulate stimulation intensity based on intended grasp force (Bouton et al., 2016; Ajiboye et al., 2017). Since this study was conducted while the subject was at rest with only visual feedback of the task, it will also be important to determine if feedback provided through restored somatosensory pathways changes the internal representation of grasp force (Flesher et al., 2016).

We have presented evidence, in a case-study, that attempted grasping of familiar objects elicits patterns of activity in M1 that are informative about implicit grasp force representations. Force-related activity was most robust during periods of active grasping and not during periods of static force application.

## Author Contributions

JD, ZT, PM, MB, RG, and JC contributed to the design of the study. JD and JW implemented the experimental paradigms. JD, JW, and SF ran experiments. JD analyzed the data. JD, SF, RG, and JC interpreted the results. All authors contributed to, and approved, the manuscript.

## Conflict of Interest Statement

The authors declare that the research was conducted in the absence of any commercial or financial relationships that could be construed as a potential conflict of interest.
